# Retraction: Long non-coding RNA SNHG16 regulates cell behaviors through miR-542-3p/HNF4α axis via RAS/RAF/MEK/ERK signaling pathway in pediatric neuroblastoma cells

**DOI:** 10.1042/BSR-2020-0723_RET

**Published:** 2024-11-11

**Keywords:** HNF4α, miR-542-3p, neuroblastoma, RAS/RAF/MEK/ERK signaling pathway, SNHG16

This article “Long non-coding RNA SNHG16 regulates cell behaviors through miR-542-3p/HNF4α axis via RAS/RAF/MEK/ERK signaling pathway in pediatric neuroblastoma cells” DOI: 10.1042/BSR20200723 is being retracted from *Bioscience Reports* at the request of the Editor-in-Chief and the Editorial Board. This follows receipt of a notification from a reader, alerting the Editorial Board to the appearance of the Western blots, which share characteristics with those found in other papers by different authors. Additionally, the Editorial Office noted multiple instances of similarities in the background details of the Western blots, specifically between:

- Figure 2F GAPDH and Figure 3I N-cadherin
- Figure 3G N-cadherin and Figure 7C RAS
- Figure 3I Vimentin and Figure 7A GAPDH
- Figure 4M HNFα and Figure 5I N-cadherin
- Figure 5G E-cadherin and Figure 5I GAPDH, and separately Figure 5G Vimentin and Figure 5I N-cadherin
- Figure 7A RAS and Figure 7C p-ERK, and separately Figure 7A p-MEK and Figure 7C RAS.

The authors have been contacted with regards to the retraction and have not responded to the journal's queries or the concerns raised. Given the extent of the issues raised, the Editorial Board stand by the decision to retract the article.

